# Dynamics of Psychopathological Disorders and Changes in the Functional Activity of Neutrophils in Mercury Intoxication

**DOI:** 10.1192/j.eurpsy.2022.1799

**Published:** 2022-09-01

**Authors:** D. Labunskiy, V. Podsevatkin, S. Kiryukhina, N. Kolmykova

**Affiliations:** Ogarev Mordovia State University, Neurology And Psychiatry, Saransk, Russian Federation

## Abstract

**Introduction:**

The overall goal was to determine the degree of mental, neuromuscular and statodynamic functions disorders resulting from toxic effect of mercury and its compounds, to study relationship between the degree of loss of professional ability to work as a result of occupational disease with the presence of complications due to concomitant diseases.

**Objectives:**

The relevance of studying the degree of violations of the main indicators of vital activity due to the toxic effect of mercury and its compounds is due to the persisting high level of loss of professional ability to work, and in some cases, and disability of this contingent. Analysis of indicators of primary and repeated disability due to occupational diseases showed that a sufficiently high number of persons recognized as disabledю

**Methods:**

We studied the dynamics of psychopathological disorders: violation of the emotional sphere, thinking, perception, attention, volitional activity and cognitive functions. The functional and metabolic activity of segmented neutrophils in the peripheral blood of 42 patients, men and women aged 43 to 64 years, and 22 healthy donors were studied.

**Results:**

Long-term exposure to chronic industrial mercury intoxication led to persistent disorders of mental functions, suppression of phagocytic activity of neutrophils up to 42.3+-4.7 per cent and inhibition of the reaction with nitro blue cytosol 4.2+-0.1 per cent..

**Conclusions:**

The revealed violations of the emotional-volitional sphere and cognitive mental functions are possibly associated with the suppression of nonspecific cellular reactions of microphages. Violations of neuroimmune interactions in mercury intoxication require further study.

**Disclosure:**

No significant relationships.

